# Supplementary material for: Heat adaptation of phage T7 under an extended genetic code
Source: Virus Evol. 2021 Dec 1;7(2):veab100. doi: 10.1093/ve/veab100 (PMC8923235; doi:10.1093/ve/veab100)
Supplement: veab100_Supp [file veab100_supp.zip › Sequencing_Analysis.zip!/Sequencing_Analysis/A2/evidence/SNP_2.html]

BRESEQ :: Evidence


| Predicted mutation | | | | | | |
| --- | --- | --- | --- | --- | --- | --- |
| evidence | seq id | position | mutation | annotation | gene | description |
| RA | NC\_001604.1 | 15,094 | G→T | intergenic (–/–) | – / – | –/– |

| Read alignment evidence... | | | | | | | | | | | |
| --- | --- | --- | --- | --- | --- | --- | --- | --- | --- | --- | --- |
|  | seq id | position | | ref | new | freq | score (cons/poly) | reads | annotation | genes | product |
| \* | NC\_001604.1 | 15,094 | 0 | G | T | 99.6% | 1047.8 / NA | 504 | intergenic (–/–) | *–/–* | –/– |
| Reads supporting (aligned to +/- strand):  **ref** base G (0/2);  **new** base T (251/249);  **total** (252/252) | | | | | | | | | | | |
| Fisher's exact test for biased strand distribution *p*-value = 4.99e-01 | | | | | | | | | | | |
| Kolmogorov-Smirnov test that lower quality scores support variant *p*-value = 1.00e+00 | | | | | | | | | | | |

Only 100 of 507 total aligned reads displayed.  

`AGAGCAGGGTGAAGAATACGTTGACGGAATGGAGTGGTGGAACTTCAACGAAGAGATGATGGACTATAACGTTCAGGACGTTGTGGTAACTAAAGCTCTCCTTGAGAAGCTACTCTCTGACAAACATTACTTCCCTCCTGAGATTGACTTTACGGACGTAGGATACACTACGTTCTGGTCAGAATCCCTTGAGGCCGTTGACATTGAACATCGTGCTGCATGGCTGCTCGCTAAACAAGAGCGCAACGGGTTCCCGTTTGACACAAAAGCAATCGAAGAGTTGTACGTAGAGTTAGCTGCTCGCCGCTCTGAGTTGCTCCGTAAATTGACCGAAACGTTCGGCTCGTGGTATCAGCCTAAAGGTGGCACTGAGATGTTCTGCCATCCGCGAACAGGTAAGCCACTACCTAAATACCCTCGCATTAAGACACCTAAAGTTGGTGGTATCTTTAAGAAGCCTAAGAACAAGGCACAGCGAGAAGGCCGTGAGCCTTGCGAACTTGATACCCGCGAGTACGTTGCTGGTGCTCCTTACACCCCAGTTGAACATGTTGTGTTTAACCCTTCGTCTCGTGACCACATTCAGAAGA  >  NC_001604.1/14796‑15385`  

                                                                                                                                                                                                                                                                                                          `|`                                                                                                                                                                                                                                                                                                     
       `ggTGAAGAATACGTTGACGGAATGGAGTGGTAGAACTTCAACGAAGAGATGATGGACTATAACGTTCAGGACGTTGTGGTAACTAAAGCTCTCCTTGAGAAGCTACTCTCTGACAAACATTACTTCCCTCCTGAGATTGACTTTACGGACGTAGGATACACTACGTTCTGGTCAGAATCCCTTGAGGACGTTGACATTGAACATCGTGCTGCATGGCTGCTCGCTAAACAAGAGCGCAACGGGTTCCCGTTTGACACAAAAGCAATCGAAGAGTTGTACGTAGAGTTAGCTTCTCGCCGct                                                                                                                                                                                                                                                                                            >  1:577867/1‑301 (MQ=255)`  
          `gaagaaTACGTTGACGGAATGGAGTGGTGGAACTTCAACGAAGAGATGATGGACTATAACGTTCAGGACGTTGTGGTAACTAAAGCTCTCCTTGAGAAGCTACTATCTGACAAACATTACTTCCCTCCTGAGATTGACTTTACGGACGTAGGATACACTACATTCTGGTCAGAATCCCTTGAGGCCGTTGACATTGAACATCGTGCTGCATGGCTGCTCGCTAAACAAGAGCGCAACGGGTTCCCGTTTGACACAAAAGCAATCGAAGAGTTGTACGTAGCGTTAGCTTCTCGCCGctctt                                                                                                                                                                                                                                                                                         >  1:386837/1‑300 (MQ=255)`  
                 `aCGTTGACTGAATGGAGTGGTGGAACTTCAACGAAGAGATGATGGACTATAACGTTCAGGACGTTGTGGTAACTAAAGCTCTCCTTGAGAAGCTACTCTCTGACAAACATTACTTCCCTCCTGAGATTGACTTTACGGACGTATGATACACTACGTTCTGGTCAGAATCCCTTGAGGCCGTTGACATTGAACATCGTGCTGCATGGCTGCTCGCTAAACAAGAGCGCAACGGGTTCCCGTTTGACACAAAAGCAATCGAAGAGTTGTACGTAGAGTTAGCTTCTCGCCGCTCTGAGTTGCt                                                                                                                                                                                                                                                                                  >  1:92851/1‑301 (MQ=255)`  
                          `gAATGGAGTGGTGGAACTTCAACGAAGAGATGATTGACTATAACGTTCAGGACGTTGTGGTAACTAAAGCTCTCCTTGAGAAGCTACTCTCTGACAAACATTACTTCCCTCCTGAGATTGACTTTACGGACGTAGGATACACTACGTTCTGGTCAGAATCCCTTGAGGCCGTTGACATTGAACATCGTGCTGCATGGCTGCTCGCTAAACAAGAGCGCAACGGGTTCCCGTTTGACACAAAAGCAATCGAAGAGTTGTACGTAGAGTTAGCTTCTCGCCGCTCTGAGTTGCTCCGTAAAtt                                                                                                                                                                                                                                                                         >  1:304003/1‑301 (MQ=255)`  
                          `gAATGGAGTGGTGGAACTTCAACGAAGAGATGATGGACTATAACGTTCAGGACGTTGTGGTAACTAAGGCTCTCCTTGAGAAGCTACTCTCTGACAAACATTACTTCCTTCCTGAGATTGACTTTACGGACGTAGGATACACTACGTTCTGTTCAGAATCCATTGAGGCCGTTGACATTTAACATCGTTCTGCATGGCTGCTCGCTAAACAAAAGCGAACCGGGTTCCCGTTTGACACAAAGCCAATCGAAGAGTTGTACGTAGAGTTGGCTTCTAGCCGCTCTGAGTTCCTCCGTAACtt                                                                                                                                                                                                                                                                         >  1:813607/1‑301 (MQ=255)`  
                              `ggAGTGGTGGAACTTCAACGAAGAGATGATGGACTATAACGTTCAGGACGTTGTGGTAACTAAAGCTCTCCTTGAGAAGCTACTCTCTGACAAACATTACTTCCCTCCTGAGATTGACTTTACGGACGTAGGATACACTACGTTCTGTTCAGAATCCTTGAAGGCCGTTGACATGGAACATCTTGCTGCATGGCGGCCGGCAAAAAAAGACCGCAAGGGGTCCCCGTTTGCAACAAAAGAAATCGAAGAGTTGTAGGAAGAGTTAGCTCCCGGCCGTTTGGAGTTGCTCCGAAAATTGAcc                                                                                                                                                                                                                                                                     >  1:701364/1‑301 (MQ=255)`  
                              `ggAGTGGTGGAACTTCAACGAAGAGATGATGGACTATAACGTTCAGGACGTTGTGGTAACTAAAGCTCTCCTTGAGAAGCTACTCTCTGACAAACATTACTTCCCTCCTGAGATTGACTTTACGGACGTAGGATACACTACGTTCTGGTCAGAATCCCTTGAGGCCGTTGACATTGAACATCGTGCTGCATGGCTGCTCGCTAAACAAGAGCGCAACGGGTTCCCGTTTGACACAAAAGCAATCGAAGAGTTGTACGTATAGTTAGCTTCTCGCCGCTCTGAGTTGCTCCGTAAATTGAcc                                                                                                                                                                                                                                                                     <  1:25558/301‑1 (MQ=255)`  
                                   `tgtgcataagagacagagagATGATGGACTATAACGTTCAGGACGTTGTGGTAACTAAAGCTCTCCTTGAGAAGCTACTCTCTGACAAACATTACTTCCCTCCTGAGATTGACTTTACGGACGTATGATACACTACGTTCTGGTCAGAATCCCTTGAGGCCGTTGACATTGAACATCGTGCGGCATGGCTGCTCGCTAAACAAGAGCGCAACGGGTTCCCGTTTGACACAAAAGCAATCGAAGAGTTGTACGTAGAGTTAGCTTCTCGACGCTCTGAGTTGCTCCGTAAATTGACCGAAAc                                                                                                                                                                                                                                                                <  1:805962/285‑1 (MQ=255)`  
                                   `ggtggAACTTCAACGAAGAGATGATGGACTATAACGTTCAGGACGTTGTGGTAACTAAAGCTCTCCTTGAGAAGCTACTCTCTGACAAACATTACTTCCCTCCTGAGATTGACTTTACGGACGTAGGATACACTACGTTCTGGTCAGAATCCCTTGAGGCCGTTGACATTGAACATCGTGCTGCATGGCTGCTCGCTAAACAAGAGCGCAACGGGTTCCCGTTTGACACAAAAGCAATCGAAGAGTTGTACGTAGAGTTAGCTTCTCGCCGCTCTGAGTTGCTCCGTAAACTgtctcttat                                                                                                                                                                                                                                                                >  1:162262/1‑293 (MQ=255)`  
                                    `gtggAACTTCAACGAAGAGATGATGGACTATAACGTTCAGGACGTTGTGGTAACTAAAGCTCTCCTTGAGAAGCTACTCTCTGACAAACATTACTTCCCTCCTGATATTGACTTTCCGGTCGTCGGATACACTACGTTCTGGTCAGAATCCATTGAGTCCGTTGACATTGAACATCGTGCTGCATGGCTGCTCGCTAAACAAGAGCGCACCGTGTTCCCGTTTGACACACAAGCAATCGACGAGTTCTACGTAGAGTTAGCTTCTCGCCGCTCTGAGTTGCTCCGTAAATTTACCGATACg                                                                                                                                                                                                                                                               >  1:153361/1‑301 (MQ=255)`  
                                         `aCTTCAACGAAGAGATGATGGACTATAACGTTCAGGACGTTGTGATAACTAAAGCTCTCCTTGAGAAGCTACTCTCTGACAAACATTACTTCCCTCCTGAGATTGACTTTACGGACGTCGGATACACTACGTTCTGGTCAGAATCCCTTGAGGCCGATGACATTGAACATCGTGCTGCATGGCTGCTCGCTAAACAATAGCGCAACGCGTTCCAGTTTGACACCCAAGCGAGCGAAGAGTTGTACGTCCAGTTATCTTCTAGCCGCCCTTAGTTGCTCCATAAATTGACCTAAACgattgt                                                                                                                                                                                                                                                          >  1:78355/1‑296 (MQ=255)`  
                                                 `aaaGAGATGATGGACTATAACGTTCAGGACGTTGTGGTAACTAAAGCTCTCCTTGAGAAGCTACTCTCTGACAAACATTACTTCCCTCCTGAGATTGACTTTACGGACGTAGGATACACTACGTTCTGGTCAGAATCCCTTGAGGCCGTTGACATTGAACATCGTGCTGCATGGCTGCTCGCTAAACAAGAGCGCAACGGGTTCCCGTTTGACACAAAAGCAATCGAAGAGTTGTACGTAGAGTTAGCTTCTCGCCGCTCTGAGTTGCTCCGTAAATTGACCGAAACGTTCGGCTCGTGGt                                                                                                                                                                                                                                                  <  1:339641/300‑1 (MQ=255)`  
                                                        `gtgggctcggagaggtgtataagagacaggTAACTAAAGCTCTCCTTGAGAAGCTACTCTCGGACAAACATTACTTCCCTCCTGAGATTGACTTTACGGACGTAGGATACACTACGTTCTGGTCAGAATCCCTTGAGGCCGTTGACATTGAACATCGTGCTGCATGGCTGCTCGCTAAACAAGAGCGCAACGGGTTCCCGTTTGACACAAAAGCAATCGAAGAGTTGTACGTAGAGTTAGCTTCTCGCCGCTCTGAGTTGCTCCGTAAATTGACCGAAACGTTCGGCTCGTGGTATCAGcc                                                                                                                                                                                                                                           <  1:537694/273‑1 (MQ=255)`  
                                                        `ggctcggagatgtgtataagagacaggtgGTAACTAAAGCTCTCCTTGAGAAGCTACTCTCTGACAAACATTACTTCCCTCCTGAGATTGACTTTACGGACGTAGGATACACTACGTTCTGGTCAGAATCCCTTGAGGCCGTTGACATTGAACATCGTGCTGCATGGCTGCTCGCTAAACAAGAGCGCAACGGGTTCCCGTTTGACACAAAAGCAATCGAAGAGTTGTACGTAGAGTTAGCTTCTCGCCGCTCTGAGTTGCTCCGTAAATTGACCGAAACGTTCGGCTCGTGGTATCAGcc                                                                                                                                                                                                                                           <  1:489430/275‑1 (MQ=255)`  
                                                             `gACTATAACGTTCAGGACGTTGTGGTAACTAAAGCTCTCCTTGAGAAGCTACTCTCTGACAAACATTACTTCCCTCCTGAGATTGACTTTACGGACGTAGGATACACTACGTTCTGGTCAGAATCCCTTGAGGCCGTTGACATTGAACATCGTGCTGCATGGCTGCTCGCTAAACAAGAGCGCAACGGGTTCCCGTTTTACACAAAAGCAATCGAAGAGTTGTACGTAGAGTTAGCTTCTCGCCGCTCTGAGTTGCTCCGTAAATTTACCGAAACGTTTGGCTCGTGGTATCAGCCTAAAg                                                                                                                                                                                                                                      >  1:514205/1‑301 (MQ=255)`  
                                                              `gctcggagatgtgtataagagacagaaCTAAAGCTCTCCTTGAGAAGCTACTCTCTGACAAACTTTACTTCCCTCCTGAGATTGACTTTACGGACGTAGGATACACTACGTTCTGGTCAGAATCCCTTGAGGCCGTTGACATTGAACATCGTGCTGCATGGCTGCTCGCTAAACAAGAGCGCAACGGGTTCCCGTTTGACACAAAAGCAATCGAAGAGTTGTACGTAGAGTTAGCTTCTCGCCGCTCTGAGTTGCTCCGTAAATTGACCGAAACGTTCGGCTCGTGGTATCAGCCTAAAgg                                                                                                                                                                                                                                     <  1:55901/276‑1 (MQ=255)`  
                                                              `agagacaggTTCAGGACGTTGTGGTAACTAAAGCTCTCCTTGAGAAGCTACTCTCTGACAAACATTACTTCCCTCCTGAGATTGACTTTACGGACGTAGGATACACTACGTTCTGGTCAGAATCCCTTGAGGCCATTGACATTGAACATCGTGCTGCATGGCTGCTCGCTAAACAAGAGCGCAACGGGTTCACGTTTGACACAAAAGCAATCGAAGAGTTGTACGTAGAGTTAGCTTCTCGCCGCTCTGAGTTGCTCCGTAAATTGACCGAAACGTTCGGCTCGTGGTATCAGCCTAAAgg                                                                                                                                                                                                                                     <  1:293016/293‑1 (MQ=255)`  
                                                              `aCTATAACGTTCAGGACGTTGTGGTAACTAAAGCTCTCCTTGAGAAGCTACTCTCTGACAAACATTACTTCCCTCCTGAGATTGACTTTACGGACGTAGGATACACTACGTTCTGGTCAGAATCCCTTGAGGCCGTTGACATTGAACATCGTGCTGCATGGCTGCTCGCTAAACAAGAGCGCAACGGGTTCCCGTTTGACACAAAAGCAATCGAAGAGTTGTACGTAGAGTTAGCTTCTCGCCGCTCTGAGTTGCTCCGTAAATTGACCGAAACGTTCGGCTCGTGGTATCAGCCTAAAgg                                                                                                                                                                                                                                     <  1:364518/301‑1 (MQ=255)`  
                                                              `aCTATAACGTTCAGGACGTTGTGGTAACTAAAGCTATCCTTGAGAAGCTACTCTCTCACAAACATTACTTCCCTCCTGAGATTGACTTTACGGACGTAGGATACACTACGTTCTGGTCAGAATCCCTTGAGGCCGTTGACATTGAACATCGTGCTGCATGGCTGCTCGCTAAACAAGAGCGCAACGGGTTCCCGTTTGACACAAAAGCAATCGAAGAGTTGTACGTAGAGTTAGCTTCTCGCCGCTCTGAGTTGCTCCGTAAATTGACCGAAACGTTCGGCTCGTGGTATCAGCCTAAAgg                                                                                                                                                                                                                                     <  1:208122/301‑1 (MQ=255)`  
                                                               `cTATAACGTTCAGGACGTTGTGGTAACTAAAGCTCTACTTGAGAAGCTACTCTCTGACAAACATTACTTCCCTCCTGAGATTGACTTTACGGACGTAGGATACACTACGTTCTGGTCAGAATCCCTTGAGGCCGTTGACATTGAACATCGTGCTGCACGGCTGCTCGCTAAACAAGAGCGCAACGGGTTCCCGTTTGACACAAAAGCAATCGAAGAGTTGTACGTAGAGTTAGCTTCTCGCCGCTCTGAGTTGCTCCGTAAATTGACCGAAACGTTCGGCTCGTGGTATCAGccctgtctc                                                                                                                                                                                                                                    >  1:634659/1‑294 (MQ=255)`  
                                                                 `ataATGTTCAGGACGTTGTGGTAACTAAAGCTCTCCTTGAGAAGCTACTCTCTGACAAACATTACTTCCCTCCTGAGATTGACTTTACGGACGTAGGATACACTACGTTCTGGTCAGAATCCCTTGAGGCCGTTGACATTGAACATCGTGCTGCATGGCTGCTCGCTAAACAAGAGCGCAACGGGTTCCCGTTTGACACAAAAGCAATCGAAGAGTTGTACGTAGAGTTAGCTTCTAGCCGCTCTGAGTTGCTCAGTAAATTGACCGAAACGTTCGGCTCGTGGTATCAGCCTAAAggctg                                                                                                                                                                                                                                  >  1:655849/1‑298 (MQ=255)`  
                                                                 `ataACGTTCAGGACGTTGTGGTAACTAAAGCTCTCCTTGAGAAGCTACTCTCTGACAAACATTACTTCCCTCCTGAGATTGACTTTACGGACGTAGGACACACTACGTTCTGGTCAGAATCCCTTGAGGCCGTTGACATTGAACATCGTGCTGCATGGCTACTCGCTAAACAAGAGCGCAACGGGTTCCCGTTTGACACAAAAGCAATCGAAGAGTTGTACGTAGCGTTAGCTTCTCGCCGCTCTGAGTTGCTCCGTAAATTGACCGAAACGTTCGGCTCGTGGTATCAGCCTAAAGGTgg                                                                                                                                                                                                                                  >  1:281487/1‑301 (MQ=255)`  
                                                                     `cGTTCAGGACGTTGTGGTAACTAAAGCTCTCCTTGAGAAGCTACTCTCTGACAAACATTACTTCCCTCCTGAGATTGACTTTACGGACGTAGGATACACTACGTTCTGGTCAGAATCCCTTGAGGCCGTTGACATTGAACATCGTGCTGCATGGCTGCTCGCTAAACAAGAGCGAAACGGGTTCCCGTTTGACACAAAAGCAATCGAAGAGTTGTACGTAGAGTTAGCTTCTCGCCGCTCTGAGTTGCTCCGTAAATTGACCGAAACGTTCGGCTCGTGGTATCAGCCTAAAGGTAGCACt                                                                                                                                                                                                                              >  1:19697/1‑301 (MQ=255)`  
                                                                      `tttCAGGACGTTGTTGTAACTAAAGCTCTCCTTGAGAAGCTACTCTCTGACAAACATTACTTCCCTCCTGAGATTGACTTTACGGACGTAGGATACACTACGTTCTGGTCAGAATCCCTTGAGGCCGTTGACATTGAACATCGTGCTGCATGGCTGCTCGCTAAACAAGAGCGCAACGGGTTCCCGTTTGACACAAAAGCAATCGAAGAGTTGTACGTAGAGTTAGCTTCTCGCCGCTCTGAGTTGCTCCGTAAATTGACCGAAACGTTCTGCTCGTGGTATCAGCCTAAAGGTGGCACTg                                                                                                                                                                                                                             <  1:673219/300‑1 (MQ=255)`  
                                                                      `gTTCAGGACGTTGTGGTAACTAAAGCTCTCCTTGAGAAGCTACTCTCTGACAAACATTACTTCCCTCCTGAGATTGACTTTACGGACGTAGGATACACTACGTTCTGGTCAGAATCCCTTGAGGCCGTTGACATTGAACATCGTGCTGCATGGCTGCTCGCTAAACAAGAGCGCAACGGGTTCCCGTTTGACACAAAAGCAATCGAAGAGTTGTACGTAGAGTTAGCTTCTCGCCGCTCTGAGTTGCTCCGTAAATTGACCGAAACGTTCGGCTCGTGGTATCAGCCTAAAGGTGGCACTg                                                                                                                                                                                                                             >  1:645184/1‑301 (MQ=255)`  
                                                                      `gTTCAGGACGTTGTGGTAACTAAAGCTCTCCTTGAGAAGCTACTCTCTGACAAACATTACTTCCCTCCTGAGATTGACTTTACGGACGTAGGATACACTACGTTCTGGTCAGAATCCCTTGAGGCCATTGACATTGAACATCGTGCTGCATGGCTGCTCGCTAAACAAGAGCGCAACGGGTTCCCGTTTGACACAAAAGCAATCGAAGAGTTTTACGTAGAGTTAGCTTCTCGCCGCCCTGAGTTGCTCCGTAATTTGACCGAAACGTTCGGCCCGTGGTATCAGCCTAAAggctgtctct                                                                                                                                                                                                                             >  1:350059/1‑293 (MQ=255)`  
                                                                                 `tgtgGTAACTAAAGCTCTCCTTGAGAAGCTACTCTCTGACAAACATTACTTCCCTCCTGAGATTGACTTTACGGACGTAGGATACACTACGTTCTGGTCAGAATCCATTGAGGCCGTTGACATTGAACATCGTGCTGCATGGCTGCTCGCTAAACAAGAGCGCAACGGGTTCCCGTTTGACACAAAAGCAATCGAAGAGTTGTACGTAGAGTTAGCTTCTCGCCGCTCTGAGTTGCTCCGTAAATTGACCGAAACGTTCGGCTCGTGGTATCAGCCTAAAGGTGGCACTGAGATGTTCTGc                                                                                                                                                                                                                  <  1:371255/301‑1 (MQ=255)`  
                                                                                  `gtgGTAACTAAAGCTCTCCTTGAGAAGCTACTCTCTGACAAACATTACTTCCCTCCTGAGATTGACTTTACGGACGTAGGATACACTACGTTCTGGTCAGAATCCCTTGAGGCCGTTGACATTGAACATCGTGCTGCATGGCTGCTCGCTAAACAAGAGCGCAACGGGTTCCCGTTTGACACAAAAGCAATCGAAGAGTTGTACGTAGAGTTAGCTTCTCGCCGCTCTGAGTTGCTCCGTAAATTGACCGAAACGTTCGGCTCGTGGTATCAGCCTAAAGGTGGCACTGAGATGTTCTGcc                                                                                                                                                                                                                 >  1:435085/1‑301 (MQ=255)`  
                                                                                   `gggTAACTAAAGCTCTCCTTGAGAAGCTACTCTCTGACAAACATTACTTCCCTCCTGAGATTGACTTTACGGACGTAGGATACACTACGTTCTGGTCAGAATCCCTTGAGGCCGTTGACATTGAACATCGTGCTGCATGGCTGCTCGCTAAACAAGAGCGCAACGGGTTCCCGTTTGACACAAAAGCAATCGAAGAGTTGTACGTAGAGTTAGCTTCTCGCCGCTCTGAGTTGCTCCGTAAATTGACCGAAACGTTCGGCTCGTGGTATCAGCCTAAAGGTGGCACTGAGATGTTCTGCCa                                                                                                                                                                                                                <  1:115437/300‑1 (MQ=255)`  
                                                                                       `aaCTAAAGCTCTCCTTGAGAAGCTACTCTCTGACAAACATTACTTCCCTCCTGAGATTGACTTTACGGACGTAGGATACACTACGTTCTGGTCAGAATCCCTTGAGGCCGTTGACATTGAACATCGTGCTGCATGGCTGCTCGCTAAACAAGAGCGCAACGGGTTCCCGTTTGACACAAAAGCAATCGAATAGTTGTACGTAGAGTTAGCTTCTCGCCGCTCTGAGTTGCTCCGTAAATTGTCCGAAACGTTCGGCTCGTGGTATCAGCCTAAAGGTGGCACTGAGATGTTCTGCCATccc                                                                                                                                                                                                            >  1:688702/1‑300 (MQ=255)`  
                                                                                        `aCTAAAGCTCTCCTTGAGAAGCTACTCTCTGACAAACATTACTTCCCTCCTGAGATTGACTTTACGGACGTAGGATACACTACGTTCTGGTCAGAATCCCTTGAGGCCGTTGACATTGAACATCGTGCTGCATGGCTGCTCGCTAAACAAGAGCGCAACGGGTTCCCGTTTGACACAAAAGCAATCGAAGAGTTGTACGTAGAGTTAGCTTCTCGCCGCTCTTAGTTGCTCCGTAAATTGACCGCAACGTTCGGCTCGTGGTATCAGCCTAAAGGTGGCACTGAGATGTTCTGCCATCcgc                                                                                                                                                                                                           >  1:227461/1‑301 (MQ=255)`  
                                                                                                   `ccTTGAGAAGCTACTCTCTGACAAACATTACTTCCCTCCTGAGATTGACTTTACGGACGTAGGATACACTACGTTCTGGTCAGAATCCCTTGAGGCCGTTGACATTGAACATCGTGCTGCATGGCTGCTCGCTAAACAAGAGCGCAACGGGTTCCCGTTTGACACAAAAGCAATCGAAGAGTTGTACGTAGAGTTAGCTTCTCGCCGCTCTGAGTTGCTCCGTAAATTGACCGAAACGTTCGGCTCGTGGTATCAGCCTAAAGGTTGCACTTAGATGTTCTGCCATCCGCGACCAGGTAAg                                                                                                                                                                                                >  1:452101/1‑301 (MQ=255)`  
                                                                                                   `ccTTGAGAAGCTACTCTCTGACAAACATTACTTCCCTCCTGAGATTGACTTTACGGACGTAGGATACACTACATTCTGGTCAGAATCCCTTGAGGCCGTTGACATTGAACATCGTGCTGCATGGCTGCTCGCTAAACAAGAGCGCAACGGGTTCCCGTTTGACACAAAAGCAATCGAAGAGTTGTACGTAGAGTTAGCTTCTCGCCGCTCTGAGTTGCTCCGTAAATTGACCGAAACGTTCGGCTCGTGGTATCAGCCTAAAGGTGGCCCTGAGATGTTCTGCCATCCGCAAAcctgtctc                                                                                                                                                                                                >  1:619748/1‑294 (MQ=255)`  
                                                                                                       `gagaAGCTACTCTCTGACAAACATTACTTCCCTCCTGAGATTGACTTTACGGACGTAGGATACACTACGTTCTGGTCAGAATCCCTTGAGGCCGTTGACATTGAACATCGTGCTGCATGGCTGCTCGCTAAACAAGAGCGCAACGGGTTCCCGTTTGACACAAAAGCAATCGAAGAGTTGTACGTAGAGTTAGCTTCTCGCCGCTCTGAGTTGCTCCGTAAATTGACCGAAACGTTCGGCTCGTGGTATCAGCCTAAAGGTGGCACTGAGATGTTCTGCCATCCGCGAACAGGTAAGCCAc                                                                                                                                                                                            <  1:320672/301‑1 (MQ=255)`  
                                                                                                           `aGCTAATCTCTGACAAACATTACTTCCCTCCTGAGATTGACTTTACGGACGTAGGATACACTACGTTCTGGTCAGAATCCCTTGAGGCCGTTGACATTTAACATCGTGCTGCATGGCTGCTCGCTAAACAAGAGCGCAACGGGTTCCCGTTTGACACAAAAGCAATCGAAGAGTTGTACGTAGAGTTAGCTTCTCGCCGCTCTGAGTTGCTCCGTAAATTGACCGAAACGTTCGGCTCGTGGTATCAGCCTAAAGGTGGCACTGAGATGTTCTGCCATCCGCGAACAGGTAAGCCACTAcc                                                                                                                                                                                        <  1:677311/301‑1 (MQ=255)`  
                                                                                                                 `gacaggACAAACATTACTTCCCTCCTGAGATTGACTTTACGGACGTAGGATACACTACGTTCTGGTCAGAATCCATTGAGGCCGTTGACATTGAACATCGTGCTGCATGGCTGCTCGCTAAACAAGAGAGCAACGGGTTCCCGTTTGACACAAAAGCAATCGAAGAGTTGTACGTAGAGTTAGCTTCTCACCACTCTGAGTTGCTCCGTAAATTGACCGAAACGTTCGGCTCGTGGTATCAGCCTAAAGGTGGCACTGAGATGTTCTGCCATCAGCGAACAGGTAAGCCACTACCTAAATa                                                                                                                                                                                  <  1:533497/296‑1 (MQ=255)`  
                                                                                                                  `taagagacagcATTACTTCCCTCCTGAGATTGACTTTACGGACGTAGGATACACTACGTTCTGGTCAGAATCCCTTGAGGCCGTTGACATTGAACATCGTGCTGCATGGCTGCTCGCTAAACAAGAGCGCAACGGGTTCCCGTTTGACACAAAAGCAATCGAAGAGTTGTACGTAGAGTTAGCTTCTCGCCGCTCTGAGTTGCTCCGTAAATTGACCGAAACGTTCGGCTCGTGGTATCAGCCTAAAGGTGGCACTGAGATGTTCTGCCATCCGCGAACAGGTAAGCCACTACCTAAATAc                                                                                                                                                                                 <  1:419972/291‑1 (MQ=255)`  
                                                                                                                  `ctctGACAAACATTACTTCCTTCCTGAGATTGACTTTACGGACGTAGGATACACTACGTTCTGGTCAGAATCCCTTGAGGCCGTTGACATTGAACATCGTGCTGCATGGCTGCTCGCTAAACAAGAGCGCAACGGGTTCCCGTTTGACACAAAAGCAATCGAAGAGTTGTACGTAGAGTTAGCTTCTCGCCGCTCTGAGTTGCTCCGTAAATTGACCGAAACGTTCGGCTCGTGGTATCAGCCTAAAGGTGGCACTGAGATGTTCTGCCATCCGCGAACAGGTAAGCCACTACCTAAATAc                                                                                                                                                                                 <  1:157329/301‑1 (MQ=255)`  
                                                                                                                  `ctctGACAAACATTACTGCCCTCCTGGGATTGACTTTACTGATGTAGGAGACACTACGTTCTGGTCAGAACCCCTTGAGGCCGTTGACAGTGAACATCGTGCTACATGGCTGCTCGCTAAACAAGAGCGCAACGGGTTCCCGTTTGACACAAAAGCAATCGAAGAGTTGTACGTAGAGTTAGCTTCTCGCCGCTCTGAGTTGCTCCGTAAATTGACCGAAACGTTCGGCTCGTGGTATCAGCCTAAAGGTGGCACTGAGATGTTCTGCCATCCGCGAACAGGTAAGCCACTACCTAAATAc                                                                                                                                                                                 <  1:362686/301‑1 (MQ=255)`  
                                                                                                                  `ctctGACAAACATTACGTCCCTCCTGAGATTGACTTTACGGACGTAGGATACACTACGTTCTGGTCAGAATCCCTTGAGGCCGTTGACATTGAACATCGTGCTGCATGGCTACTCGCTAAACAAGAGCGCAACGGGTTCCCGTTTGACACAAAAGCAATCGAAGAGTTGTACGTAGAGTTAGCTTCTCGCCGCTCTGAGTTGCTCCGTAAATTGACCGAAACGTTCGGCTCGTGGTATCAGCCTAAAGGTGGCACTGAGATGTTCTGCCATCCGCGAACAGGTAAGCCACTACCTAAATAc                                                                                                                                                                                 <  1:397080/301‑1 (MQ=255)`  
                                                                                                                    `cttaCAAACATTACTTCCCTCCTGAGATTGACTTTACGGACGTAGGATACACTACGTTCTGGTCAGAATCCCTTGAGGCCGTTGACATTGAACATCGTGCTGCATGGCTGCTCGCTAAACAAGAGCGCAACGGGTTCCCGATTGACACAAAAGCAATCGAAGAGTTGTCCGTAGAGTTAGCTTCTCGACGCTCTGAGTTGCTCCGTAAATTGACCGAAACGTTCGGCTCGTGGTATCAGCCTAAAGGTGGCACTGAGATGTTCTGCCATCCGCGAACAGGTAAGCCACTaacctgtctctt                                                                                                                                                                               >  1:798706/4‑290 (MQ=255)`  
                                                                                                                      `gACAAACATTACTTCCCTCCTGAGATTGACTTTACGGACGTAGGATACACTACGTTATGGTCAGAATCCCTTGAGGCCGTTGACATTGAACATCGTGCTGCATGGCTGCTCGCTAAACAAGAGCGCAACGGGTTCCCGTTTGACACAAAAGCAATCGAAGAGTTGTACGTAGAGTTAGCTTCTCGCCGCTCTGAGTTGCTCCGTAAATTGACCGAAACGTTCGGCTCGTGGTATCAGCCTAAAGGTGGCACTGAGATGTTCTGCCATCCGCGAACAGGTAAGCCACTACCTAAATACCCTc                                                                                                                                                                             >  1:667829/1‑301 (MQ=255)`  
                                                                                                                           `aCATTACTTCCCTCCTGAGATTGACTTTACGGACGTAGGATACACTACGTTCTGGTCAGAATCCCTTGAGGCCGTTGACATTGAACATCGTGCTGCATGGCTGCTCGCTAAACAAGAGCGCAACGTGTTCCCGTTTGACACAAAAGCAATCGAAGAGTTGTACGTAGAGTTAGCTTCTCGCCGCTCTGAGTTGCTCCGTAAATTGACCGAAACGTTCGGCTCGTGGTATCAGCCTAAAGGTTGCACTGAGATGTTCTGCCATCCGCGAACAGGTAAGCCACTACCTAAATAcctgtctctt                                                                                                                                                                        >  1:474270/1‑293 (MQ=255)`  
                                                                                                                           `aCATTACTTCCCTCCTGAGATTGACTTTACGGACGTAGGATACACTACGTTCTGGTCAGAATCCCTTGAGGCCGTTGACATTGAACATCGTGCTGCATGGCTGCTCGCTAAACAAGAGCGCAACGGGTTCCCGTTTGACACAAAAGCAATCGAAGAGTTGTACGTAGAGTTAGCTTCTCGCCGCTCTGAGTTACTCCGTAAATTGACCGAAACGTTCGGCTCGTGGTATCAGCCTAAATGTGGCACTGAGATGTTCTGCCATCCGCGAACAGGTAAGCCACTACCTAAATACCCTCGCAtt                                                                                                                                                                        >  1:256687/1‑301 (MQ=255)`  
                                                                                                                            `cATTACTTCCCTCCTGAGATTGACTTTACGGACGTAGGATACACTACGTTCTGGTCAGAATCCCTTGAGGCCGTTGACATTGAACATCGTGCTGCATGGCTGCTCGCTAAACAAGAGCGCAACGGGTACCCGTTTGACACAAAAGCAATCGAAGAGTTGTACGTAGAGTTAGCTTCTCGCCGCTCTGAGTTGCTCCGTAAATTGACCGAAACGTTCGGCTCGTGGTATCAGCCTAAAGGTGGCACTGAGATGTTCTGCCATCCGTGAACATGTAAGCCAcctgtctcttatacacatctcc                                                                                                                                                                       >  1:136873/1‑280 (MQ=255)`  
                                                                                                                            `cATTACTTCCATCCTGAGATTGACTTTACGGACGTAGGATACACTACGTTCTGGTCAGAATCCATTGAGGCCGTTGACATTGAACATCGTGCTGCATGGCTGCTCGCTAAACAAGAGCGCAACGGGTTCCCGTTTGACACAAAAGCAATCGAAGAGTTGTACGTAGAGTTAGCTTCTCGCCGCTCTGAGTTGCTACGTAAATTGACCGAAACGTTCGGATCGTGGTATCAGCCTAAAGGTGGAACTGAGATGTTCTGCCATCCGCGAACAGGTAAGCCACTACCTAAATACCCTCGCATTa                                                                                                                                                                       >  1:768087/1‑301 (MQ=255)`  
                                                                                                                              `ttACTTCCCTCCTGAGATTGACTTTACGGACGTAGGATACACTACGTTCTGGTCAGAATCCCTTGAGGCCGTTGACATTGAACATCGTGCTGCATGGCTGCTCGCTAAACAAGAGCGCAACGGGTTCCCGTTTGACACAAAAGCAATCGAAGAGTTGTACGTAGAGTTAGCTTCTCGCCGCTCTGAGTTGCTCCGTAAATTGACCGAAACGTTCGGCTCGTGGTATCAGCCTAAATGTGGCACTTAGATGTTCTGCCATCCGCGAACAGGTAAGCCACTACCTAAATACCCTCGCATTAAg                                                                                                                                                                     <  1:753472/301‑1 (MQ=255)`  
                                                                                                                                `agagacagcctGAGATTGACTTTACTGACGTAGGATACACTACGTTCTGGTCAGAATCCCTTGAGGCCGTTGACATTGAACATCGTGCTGCATGGCTGCTCGCTAAACAAGAGCGCAACGGGTTCCCGTTTGACACAAAAGCAATCGAAGAGTTGTACGTAGAGTTAGCTTCTCGCCGCTCTGAGTTGCTCCGTAAATTGACCGAAACGTTCGGCTCGTGGTATCAGCCTAAAGGTGGCACTGAGATGTTCTGCCATCCGCGAACAGGTAAGCCACTACCTAAATACACTCGCATTAAGac                                                                                                                                                                   <  1:565623/293‑1 (MQ=255)`  
                                                                                                                                        `cctGAGATTGACTTTACGGACGTAGGATACACTACGTTCTGGTCAGAATCCCTTGAGGCCGTTGACATTGAACATCGTGCTGCATGGCTGCTCGCTAAACAAGAGCGCAACGGGTTCCCGTTTGACACAAAAGCAATCGAAGAGTTGTACGTAGAGTTAGCTTCTCGCCGCTCTGAGTTGCTCCGTAAATTGACCGAAACGTTCGGCTCGTGGTATCAGCCTAAAGGTGGCACTGAGATGTTCTGCCATCCGCGAACAGGTAAGCCACTACCTAAATACCCTTGCATTAAGACACCTAAAg                                                                                                                                                           >  1:521295/1‑301 (MQ=255)`  
                                                                                                                                         `gctcggagatgtgtataagagacagATACACTACGTTCTGGTCAGAATCCCTTGAGGCCGTTGACATTGAACATCGTGCTGCATGGCTGCTCGCTAAACAAGAGCGCAACGGGTTCCCGTTTGACACAAAAGCAATCGAAGAGTTGTACGTAGAGTTAGCTTCTCGCCGCTCTGAGTTGCTCCGTAAATTGACCGAAACGTTCGGCTCGTGGTATCAGCCTAAAGGTGGCACTGAGATGTTCTGCCATCCGCGAACAGGTAAGCCACTACCTAAATACCCTCGCATTAAGACACCTAAAGt                                                                                                                                                          <  1:71224/277‑1 (MQ=255)`  
                                                                                                                                         `ctGAGATTGACTTTACGGACGTAGGATACACTACGTTCTGGTCAGAATCCCTTGAGGCCGTTGACATTGAACATCGTGCTGCATGGCTGCTCGCTAAACAAGAGCGCAACGGGTTCCCGTTTGACACAAAAGCAATCGAAGAGTTGTACGTAGAGTTAGCTTCTCGCCGCTCTGAGTTGCTCCGTAAATTGACCGAAACGTTCGGCTCGTGGTATCAGCCTAAAGGTGGCACTGAGATGTTCTGCCATCCGCGAACAGGTAAGCCACTACCTAAATACCCTCGCATTAAGACACCTAAAGt                                                                                                                                                          <  1:241177/301‑1 (MQ=255)`  
                                                                                                                                         `aaagagacagcTTTACGGACGTAGGATACACTACGTTCTGGTCAGAATCCCTTGAGGCCGTTGACATTGAACATCGTGCTGCATGGCTGCTCGCTAAACAAGAGCGCAACGGGTTCCCGTTTGACACAAAAGCAATCGAAGAGTTGTACGTAGAGTTAGCTTCTCGCCACTCTGAGTTGCTCCGTAAATTGACCGAAACGTTCGGCTAGTGGTATCAGCCTAAAGGTGGCACTGAGATGTTCTGCCATCCGCGAACAGGTAAGCCACTACCTAAATACCCTCGCATTAAGACACCTAAAGt                                                                                                                                                          <  1:190351/291‑1 (MQ=255)`  
                                                                                                                                          `tGAGATTGACTTTACGGACGTAGGATACACTACGTTCTGGTCAGAATCCCTTGAGGCCGTTGACATTGAACATCGTGCTGCATGGCTGCTCGCTAAACAAGAGCGCAACGGGTTCCAGTTTGACACAAAAGCAATCGAAGAGTTGTACGTAGAGTTAGCTTCTCGCCGCTCTGAGTTGCTCCGTAAATTGACCGAAACGTTCGGCTCGTGGTATCAGCCTAAAGGTGGCACTGAGATGTTCTGCCATCCGCGAACAGGTAAGCCACTACCTAAATACCCTCGCATTAAGACACCTAAAGtt                                                                                                                                                         <  1:41586/301‑1 (MQ=255)`  
                                                                                                                                           `gagaTTGACTTTACGGACGTAGGATACACTACGTTCTGGTCAGAATCCCTTGAGGCCGTTGACATTGAACATCGTGCTGCATGGCTGCTCGCTAAACAAGAGCGCAACGGGTTCCCGTTTGACACAAAAGCAATCGAAGAGTTGTACGTAGAGTTAGCTTCTCGCCGCTCTGAGTTGCTCCGTAAATTGACCGAAACGTTCGGATCGTGGTATCAGCCTAAAGGTGGCACTAAGATGTTCTGCCATCCGCGAACAGGTAAGCCACTACCTAAAtctgtctcttttacacatctccgagccc                                                                                                                                                        >  1:170550/1‑274 (MQ=255)`  
                                                                                                                                               `ggggctcggagatgtgtataagagacaggTTCTGGTCAGAATCCCTTGAGGCCGTTGACATTGAACATCGTGCTGCATGGCTGCTCGCTAAACAAGAGCGCAACGGGTTCCCGTTTGACACAAAAGCAATCGAATAGTTGTACGTACAGTTAGCTTCTCGCCGCTCTGAGTTGCTCCGTAAATTGACCGAAACGTTCGGCTCGTGGTATCAGCCTAAAGGTGGCACTGAGATGTTCTGCCATCCGCGAACAGGTAAGCCACTACCTAAATACCCTCGCATTAAGACACCTAAAGTtggtgg                                                                                                                                                    <  1:89304/273‑1 (MQ=255)`  
                                                                                                                                                          `gatgTAGGATACACTACGTTCTGGTCAGAATCCCTTGAGGCCGTTGACATTGAACATCGTGCTGCATGGCTGCTCGCTAAACAAGAGCTCAACGGGTTCCCGTTTGACACAAAAGCAATCGAAGAGTTGTACGTAGAGTTAGCTTCTCGCCGCTCTGAGTTGCTCCGTAAATTGACCGAAACGTTCGGCTCGTGGTATCAGCCTAAAGGTGGCACTGAGATGTTCTGCCATCCGCGAACAGGTAAGCCACTACCTAAATACCCTCGCATTAAGACACCTAAAGTTGGTGGTATCTTTaagc                                                                                                                                         >  1:585851/4‑300 (MQ=255)`  
                                                                                                                                                          `gACGTAGGATACACTACGTTCTGGTCAGAATCCCTTGAGGCCGTTGACATTGAACATCGTGCTGCATGGCTGCTCGCTAAACAAGAGCGCAACGGGTTCCCGTTTGACACAAAAGCAATCGAAGAGTTGTACGTAGAGTTAGCTTCTCTCCGCTCTGAGTTGCTCCGTAAATTGACCGAAACGTTCGGCTCGTGGTATCAGCCTAAAGGTGGCACTGAGATGTTCTGCCATCCGCGAACAGGTAAGCCACTACCTAAATACCCTCGCATTAAGACCCCTAAAGTTGGTGGTATCTGTaaga                                                                                                                                         >  1:273863/1‑301 (MQ=255)`  
                                                                                                                                                          `gACGTAGGATACACTACGTTCTGGTCAGAATCCCTTGAGGCCGTTGACATTGAACATCGTGCTGCATGGCTGCTCGCTAAACAAGAGCGCAACGGGTTCCCGTTTGACACAAAAGCAATCGAAGAGTTGTACGTAGAGTTAGCTTCTCGCCGCTCTGAGTTGCTCCGTAAATTTACCGAAACGTTCGGCTCGTGGTATCAGCCTAAAGGTGGCACTGAGATGTTCTGCCATCCGCGAACAGGTAAGCCACTACCTAAATACCCTCGCATTAAGACACCTAAAGTTGGTGGTATCTTTaaga                                                                                                                                         <  1:128787/301‑1 (MQ=255)`  
                                                                                                                                                          `gACGTAGGATACACTACGTTCTGGTCAGAATCCCTTGAGGCCGTTGACATTGAACATCGTGCTGCATGGCTGCTCGCTAAACAAGAGCGCAACGGGTTCCCGTTTGACACAAAAGCAATCGAAGAGTTGTACGTAGAGTTAGCTTCTCGCCGCTCTGAGTTGCTCCGTAAATTGACCGAAACGTTCGGCTCGTGGTATCAGCCTAACGGTGGCACTGAGATGTTCTGCCATCCGCGAACAGGTAAGCCACTACCTAAATACCCTCGCATTAAGACACCTAAAGTTGGTGGTATCTTTaagc                                                                                                                                         >  1:466673/1‑300 (MQ=255)`  
                                                                                                                                                               `aGGATACACTACGTTCTGGTCAGAATCCCTTGAGGCCGTTGACATTTAACATCGTGCTGCATGGCTGCTCGCTAAACAATAGCGCAACGGGTTCCCGTTTGACACAAAAGCAATCGAAGAGTTGTACGTAGAGTTAGCTTCTCTCCGCTCTGAGTTGCTCCGTAAATTGACCGAAACGTTCGGCTCGTGGTATCAGCCTAAAGGTGGCACTGAGATGTTCTGCCATCCGCGAACAGGTAAGCCACTACCTAAATACCCTTGCATTAAGACACCTAAAGTTGGTGGTATCTTTAAGAAGCCt                                                                                                                                    >  1:662371/1‑301 (MQ=255)`  
                                                                                                                                                                     `ggagatgtgtataagagacagccTTGAGGCCGTTGACATTGAACATCGTGCTGCATGGCTGCTCGCTAAACAAGAGCGCAACGGGTTCCCGTTTGACACAAAAGCAATCGAAGAGTTGTACGTAGAGTTAGCTTCTCGCCGCTCTGAGTTGCTCCGTAAATTGACCGAAACGTTCGGCTAGTGGTATCAGCCTAAAGGTGGCACTGAGATGTTCTGCCATCCGCGAACAGGTAAGCCACTACCTAAATACCCTCGCATTAAGACACCTAAAGTTGGTGGTATCTTTAAGAAGCCTAAGAAc                                                                                                                              <  1:264692/280‑1 (MQ=255)`  
                                                                                                                                                                     `cacTACGTTCTGGTCAGAATCCCTTGAGGCCGTTGACATTGAACATCGTGCTGCATGGCTGCTCGCTAAACAAGAGCGCAACGGGTTCCCGTTTGACACAAAAGCAATCGAAGAGTTGTACGTAGAGTTAGCTTCTCGCCGCTCTGAGTTGCTCCGTAAATTGACCGAAACGTTCGGCTCGTGGTATCAGCCTAAAGGTGGCACTGAGATGTTCTGCCATCCGCGAACAGGTAAGCCACTACCTAAATACCCTCGCATTAAGACACCTAAAGTTGGTGGTATCTTTAAGAAGCCTACGAAc                                                                                                                              >  1:494558/1‑301 (MQ=255)`  
                                                                                                                                                                          `ggctcggagatgtgtataagagacaggTTGACATTGAACATCGTGCTGCATTGCTGCTCGCTAAACAAGAGCGCAACGGGTTCCCGTTTGACACAAAAGCAATCGAAGAGTTGTACGTAGAGTTAGCTTCTCGCCGCTCTGAGTTGCTCCGTAAATTGACCGAAACGTTCGGCTCGTGGTATCAGCCTAAAGGTGGCACTGAGATGTTCTGCCATCCGAGAACAGGTAAGCCACTACCTAAATACCCTCGCATTAAGACACCTAAAGTTGGTGGTATCTTTAAGAAGCCTAAGAACAAGGc                                                                                                                         <  1:529597/275‑1 (MQ=255)`  
                                                                                                                                                                          `cGTTCTGGTCAGAATCCCTTGAGGCCGTGGACATTGAACATCGTGCTGCATGGCTGCTCGCTAAACAAGAGCGCAACGGTTTCCCGTTTGACACAAAAGCAATCGAAGAGTTGTACGTAGAGTTAGCTTCTCGCCGCTCTGAGTTGCTCCGTAAATTGACCGAAACGTTCGGCTCGTGGTATCAGCCTAAAGGTGGCACTGAGATGTTCTGCCATACGCGAACAGGTAAGCCACTACCTAAATACCCTCGCATTAAGACACCTAAAGTTGGTGGTATCTTTAAGAAGCCTAAGAACAAGGc                                                                                                                         <  1:407612/301‑1 (MQ=255)`  
                                                                                                                                                                          `agTTCTGGTCATAAGACCTTGAGGCCGTTGACATTGAACATCGAGCTGCATGGCTGCTCGCTAAACAAGAGCGCAACGGGTTCCCGTTTGACACAAAAGCAATCGAAGAGTTGTACGTAGAGTTAGCTTCTCGCCGCTCTGAGTTGCTCCGTAAATTGACCGAAACGTTCGGCTCGTGGTATCAGCCTAAAGGTGGCACTGAGATGTTCTGCCATCCGCGAACAGGTAAGCCACTACCTAAATACCCTCGCATTAGGACACCTAAAGTTGGTGGTATCTTTAAGAAGCCTAAGAACAAGGc                                                                                                                         <  1:84690/300‑1 (MQ=255)`  
                                                                                                                                                                            `ttCTGGTCAGAATCCGTGGAGTCCGTGGACATTGAACATCGTGCTGCATGGCTGCTCGCTAAACAAGAGCGCAATGGGTTCCCGTTTGACACAAAAGCAATCGAAGAGTTGTACGTAGAGTTAGCTTCTCGCCGCTCTGAGTTGCTCCGTAAATTGACCGAAATGTTCGGCTCGTGGTATCAGCCTAAAGGTGGCACTGAGATGTTCTGCCATCCGCGAACAGGTAAGCCACTACCTAAATACCCTCGCATTAAGACACCTAAAGTTGGTGGTATCTTTAAGAAGCCTAAGAACAAGGcac                                                                                                                       <  1:327945/301‑1 (MQ=255)`  
                                                                                                                                                                                    `aGAATCCCTTGAGGCCGTTGACATTGAACATCGTGCTGCATGGCTGCTCGCTAAACAAGAGCGCAACGGGTTCCCGTTTGACACAAAAGCAATCGAAGAGTTGTATGTAGAGTTAGCTTCTCGCCGCTCTGAGTTGCTCCGTAAATTGACCGAAACGTTCGGCTCGTGGTATCAGCCTAAAGGTGGCACTGAGATGTTCTGCCATCCGCGAACAGGTAAGCCACTACCTAAATACCCTCGCATTAAGACACCTAAAGTTGGTGGTATCTTTAAGAAGCCTAAGAACAAGGCACAGCGAGaa                                                                                                               <  1:571355/301‑1 (MQ=255)`  
                                                                                                                                                                                     `gAATCCCTTGAGGCCGTTGACATTGAACATCGTGCTGCATGGCTGCTCGCTAAACAAGAGCGCAACGGGTTCCCGTTTGACACAAAAGCAATCGAAGAGTTGTACGTAGAGTTAGCTTCTCGCCGCTCTGAGTTGCTCCGTAAATTGACCGAAACGTTCGGCTCGTGGTATCAGCCTAAAGGTGGCACTGAGATGTTCTGCCATCCTCGAACAGGTAAGCCACTACCTAAATACCCTCGCATTAAGACACCTAAAGTTGGTGGTATCTTTAAGAAGCCTAAGAAcctgtctcttatacaca                                                                                                              >  1:461558/1‑285 (MQ=255)`  
                                                                                                                                                                                             `aataagagacagcATTGAACATCGTGCTGCATGGCTGCTCGCTAAACAAGAGCGCAACGGGTTCCCGTTGGACACAAAAGAAATCGAAGAGTTGTACGTAGAGTTAGCTTCTCGCCGCTCTGAGTTGCTACGTAAATTGACCGAAACGTTCGGCTCGTGGTATCAGCCTAAAGGTGGCACTGAGATGTTCTGCCATCCGCGAACAGGTAAGCCAATACCTAAATACCCTCGCATTAAGACACCTAAAGTTGGTGGTATCTTTAAGAAGCCTAAGAACAAGGCACAGCGAGAAGGCCGTGAg                                                                                                      <  1:608510/289‑1 (MQ=255)`  
                                                                                                                                                                                              `ggtgCCGTTGACATTGAACATCGTGCTGAATGGCTGCTCGCTAAACAAGAGCGCAACGGGTTCCCGTTTGACACAAAAGCAATCTAAGAGTTGTACGTATTGTTAGCTTCTCGCCTCTCTGAGTTGCTCCGTAAATTGACCGAAACGTTCGGCTCGTGGTATCAGCCTAAAGGTGGCACTGAGATGTTCTGCCATCCGCGAACAGGTAAGCCACTACCTAAATACCCTCGCATTAAGACACCTAAAGTTGGTGGTATCTTTAAGAAGCCTAAGAACAAGGCACAGCGAGAAGGCCGTGAGc                                                                                                     <  1:613918/298‑1 (MQ=255)`  
                                                                                                                                                                                                   `cGTTGACATTGAACATCGTGCTGCATGGCTGCTCGCTAAACAAGAGCGCAACTGGTTCCCGTTTGACACAAAAGCAATCGAAGAGTTGTACGTAGAGTTAGCTTCTCGCCGCTCTGAGTTGCTCCGTAAATTGACCGAAACGTTCGGCTCGTGGTATCAGCCTAAAGGTGGCACTGAGATGTTCTGCCATCCGCTAACAGGTAAGCCACTACCTAAATACCCTCGCATTAAGACACCTAAAGTTGGTGGTATCTTTAAGAAGCCTAAGAACAAGGAACAGCGAGAAGGCCGTAAGCCTTGc                                                                                                >  1:552910/1‑301 (MQ=255)`  
                                                                                                                                                                                                        `aCATTGAACATCGTTCTGCATGGCTGCTCGCTAAACAAGAGCGCAACGGGTTCCCGTTTGACACAAAAGCAATCGAAGAGTTGTACGTAGAGTTAGCTTCTCGCCGCTCTGAGTTGCTCCGTAAATTGACCGAAACGTTCGGCTCGTGGTATCAGCCTAAAGGTGGCACTGAGATGTTCTGCCATCCGCGAACAGGTAAGCCACTACCTAAATACCCTCGCATTAAGACACCTAAAGTTGGTGGTATCTTTAAGAATCCTAAGAACAAGGCACAGCGAGAAGGCCGTGAGCCTTGCGAACt                                                                                           >  1:403842/1‑301 (MQ=255)`  
                                                                                                                                                                                                        `aCATTGAACATCGTGCTGCATGGCTGCTCGCTAAACAAGAGCGCAACGGGTTCCCGTTTGACACAAAAGCAATCGAAGAGTTGTACGTAGAGTTAGCTTCTCGCCGCTCTGAGTTGCTCCGTAAATTGACCGAAACGTTCGGCTCGTTGTATCATCCTAAATGTGGCACTGAGATGTTCTGCCATCCGCGAACAGGTAATCCACTACCTAAATACCCTCGCATTAAGACACCTAAAGTTGGTGGTATCTTTAAGAAGCCTAAGAACAAGGcctgtctcttatacacatctccgagcccacg                                                                                           >  1:596478/1‑271 (MQ=255)`  
                                                                                                                                                                                                             `gAACATCGTGCTGCATGGCTGCTCGCTAAACAAGAGCGCAACGGGTTCCCGTTTGACACAAAAGCAATCGAAGAGTTGTACGTAGAGTTAGCTTCTCGCCGCTATGAGTTGCTCCGTAAATTGACCGAAACGTTCGGCTCGTGGTATCAGCCTAAAGGTGGCACTGAGATGTTCTGCCATCCGCGAACAGGTAAGCCACTACCTAAATACCCTCGCATTAAGACACCTAAAGTTGGTGGTATCTTTAAGAAGCCTAAGAACAAGGCACAGCGAGAAgctgtctcttatacacatctccgag                                                                                      >  1:786265/1‑277 (MQ=255)`  
                                                                                                                                                                                                               `aCATCGTGCTGCATGTCTGCTCTCTAAACAAGGGCGCAACGGGTTCCCTTTTGACACAAAAGCAATCGAAGAGTTGTACGTAGAGTTAGCTTCTCGCCGCTCTGAGTTGCTCCGTAAATTGACCGAAACGTTCGGCTCGTGGTATCAGCCTAAAGGTGGCACTGAGATGTTCTGCCATCCGCGAACAGGTAAGCCACTACCTAAATACCCTCGCATTAAGACACCTAAAGTTGGTGGTATCTTTAAGAAGCCTAAGAACAAGGCACAGCGAGAAGGCCGTGAGCCTTGCGAACTTGATAcc                                                                                    <  1:443754/301‑1 (MQ=255)`  
                                                                                                                                                                                                                      `gatgtgtataagagacagaaaCAAGAGCGCAACGGGTTCCCGTTTGACACAAAAGCAATCGAAGAGTTGTACGTAGAGTTAGCTTCTCGCCGCTCTGAGTTGCTCCGTAAATTGACCGAAACGTTCGGCTCGTGGTATCAGCCTAAAGGTGGCACTGAGATGTTCTGCCATCCGTGAACAGGTAAGCCACTACCTAAATACCCACGCATTAAGACACCTAAAGTTGGTGGTATCTTTAAGAAGCCTAAGAACAAGGCACAGCGAGAAGGACGTGAGCCTTGCGAACTTGATACCCGCGAGt                                                                             <  1:178823/283‑1 (MQ=255)`  
                                                                                                                                                                                                                          `aagccaggctCGCTAAACAAGAGCGCAACGGGTTCCCGTTTGACACAAAAGCAATCGAAGAGTTGTACGTAGAGTTAGCTTCTCGCCGCTCTGAGTTGCTCCGTAAATTGACCGAAACGGTCGGCTCGTGGTATCAGCCTAAAGGTGGCACTGAGATGTTCTGCCATCCGCGAACAGGTAAGCCACTACCTAAATACCCTCGCATTAAGACACCTAAAGTTGGTGGTATCTTTAAGAAGCCTAAGAACAAGGCACAGCGAGAAGGCCGTGAGCCTTGCGAACTTGATACCCGCGAGTACGt                                                                         <  1:480959/294‑1 (MQ=255)`  
                                                                                                                                                                                                                             `ggCTGCTCGCTAAACAAGAGCGCAACGGGTTCCCGTTTGACACAAAAGCAATCGAAGAGTTGTACGTAGAGTTAGCTTCTCGCCGCTCTGAGTTGCTCCGTAAATTGACCGAAACGTTCGGCTCGTGGTATCAGCCTAAAGGTGGCACTGAGATGTTCTGCCATCCGCGAACAGGTAAGCCACTACCTAAATACCCTCGCATTAAGACACCTAAAGTTGGTGGTATCTTTAAGAAGCCTAAGAACAAGGCACAGCGAGAAGGCCGTGAGCCTTGCGAACTTGATACCCGCGAGTACGTTGc                                                                      <  1:395064/301‑1 (MQ=255)`  
                                                                                                                                                                                                                                `ggctCGCTAAACAAGAGCGCAACGGGTTCCCGTTTGACACAAAAGCAATCGAAGAGTTGTACGTAGAGTTAGCTTCTCGCCGCTCTGAGTTTCTCCGTAAATTGACCGAAACGTTCGGCTCGTGGTATCAGCCTAAAGGTGGCACTGAGATGTTCTGCCATCCGCGAACATGTAAGCCACTACCTAAATACCCTCGCATTAAGACACCTAAAGTTGGTGGTATCTTTAAGAAGCCTAAGAACAAGGCACAGCGAGAAGGCCGTGAGCCTTGCGAACTTGATACCCGCGAGTACGTTGCTgg                                                                   <  1:287080/300‑1 (MQ=255)`  
                                                                                                                                                                                                                                  `agcGCTAAACAAGAGCGCAACGGGTTCCCGTTTGACACAAAAGCAATCGAAGAGTTGTACGTAGAGTTAGCTTCTCGCCGCTCTGAGTTGCTCCGTAAATTGACCGAAACGTTCGGCTCGTGGTATCAGCCTAAAGGTGGCACTGAGATGTTCTGCCATCCGCGAACAGGTAAGCCACTACCTAAATACCCTCGCATTAAGACACCTAAAGTTGGTGGTATCTTTAAGAAGCCTAAGAACAAGGCACAGCGAGAAGGCCGTGAGCCTTGCGAACTTGATACCCGCGAGTACGTTGCTGGTg                                                                 <  1:428196/299‑1 (MQ=255)`  
                                                                                                                                                                                                                                   `tCGCTAAACAAGAGCGCAACGGGTTCCCGTTTGACACAAAAGCAATCGAAGAGTTGTACGTAGAGTTAGCTTCACGCCGCTCTGAGTTGCTCCGTAAATTGACCGAAACGTTCGGCTCGTGGTATCAGCCTAAAGGTGGCACTGAGATGTTCTGCCATCCGCGAACAGGTAAGCCACTACCTAAATACCCTCGCATTAAGACACCTAAAGTTGGTGGTATCTTTAAGAAGCCTAAGAACAAGGCACAGCGAGAAGGCCGTGAGCCTTGCGAACTTGATACCCGCTAGTACGTTGCTGGTGc                                                                <  1:718437/301‑1 (MQ=255)`  
                                                                                                                                                                                                                                    `cGCTAAACAAGAGCGAAACGGGTTCCCGTTTGACACAAAAGCAATCGAAGAGTTGTACGTAGAGTTAGCTTCTCGCCGCTCTGAGTTGCTCCGTAAATTGACCGAAACGTTCGGCTCGTGGTATCAGCCTAAAGGTGGCACTGAGATGTTCTGCCATCCGCGAACAGGTAAGCCACTACCTAAATACCCTCGCATTAAGACACCTAAAGTTGGTGGTATCTTTAAGAAGCCTAAGAACAAGGAACAGCGAGAAGGCCGTGAGCCTTGCGAACTTGATACCCGCGAGTACGTTGCTTGTGCt                                                               >  1:308523/1‑301 (MQ=255)`  
                                                                                                                                                                                                                                      `cTAAACAAGAGCGCAACGGGTTCCCGTTTGACACAAAAGCAATCGAAGAGTTGTACGTAGAGTTAGCTTCTCGCCGCTCTGAGTTGCTCCGTAAATTGACCGAAACGTTCGGCTCGTGGTATCAGCCTAAAGGTGGCACTGAGATGTTCTGCCATCCGCGAACAGGTAAGCCACTACCTAAATACCCTCGCATTAAGACACCTAAAGTTGGTGGTATCTTTAAGAAGCCTAAGAACAAGGCACAGCGAGAAGGCCGTGAGCCTTGCGAACTTGATACCCGCGAGTACGTTGCTGGTTCTcc                                                             >  1:747695/1‑301 (MQ=255)`  
                                                                                                                                                                                                                                        `aaaCAAGAGCGCAACGGGTTCCCGTTTGACACAAAAGCAATCGAAGAGTTGTACGTATAGTTAGCTTCTTGCCGCTCTGAGTTGCTCCGTAAATTGACCGAAACGTTCGGCTCGTGGTATCAGCCTAAAGGTGGCACTGAGATGTTCTGCCATCCGCGAACAGGTAAGCCACTACCTAAATACCCTCGCATTAAGACACCTAAAGTTGGTGGTATCTTTAAGAAGCCTAAGAACAAGGCACAGCGAGAAGGCCGTGAGCCTTGCGAACTTGATACCCGCAAGTAcctgtctcttatacacc                                                           >  1:150150/1‑285 (MQ=255)`  
                                                                                                                                                                                                                                          `tgtgtataagagacagTTCCCGTTTGACACAAAAGCAATCGAAGAGTTGTACGTAGAGTTAGCTTCTCGCCGCTCTGAGTTGCTCCGTAAATTGACCGAAACGTTCGGCTCGTGGTATCAGCCTAAAGGTGGCACTGAGATGTTCTGCCATCCGCGAACAGGTAAGCCACTACCTAAATACCCTCGCATTAAGACACCTAAAGTTGGTGGTATCTTTAAGAAGCCTAAGAACAAGGCACAGCGAGAAGGCCGTGAGCCTTGCGAACTTGATACCCGCAAGTACGTTGCTGGTGCTCCTTac                                                         <  1:658427/286‑1 (MQ=255)`  
                                                                                                                                                                                                                                             `atggcgcAACGGGTTCCCGTTTGAAACAAAAGCAATCGAAGAGTTGTACGTAGAGTTAGCTTCTCGCCGCTCTGAGTTGCTCCGTAAATTGACCACAACGTTCGGCTCGTGGTATCAGCCTAAAGGTGGCACTGAGATGTTCTGCCATCCGCGAACAGGTAAGCCACTACCTAAATACCCTCGCATTAAGACACCTAAAGTTGGTGGTATCTTTAAGAAGCCTAAGAACAAGGCACAGCGAGAAGGCCGTGAGCCTTGCGAACTTGATACCCGCGAGTACGTTGCTGGTGCTCCTTACAcc                                                      <  1:543261/298‑1 (MQ=255)`  
                                                                                                                                                                                                                                               `agCGCAACGGGTTCCCGTTTGACACAAAAGCAATCGAAGAGTTGTACGTAGAGTTAGCTTCTCGCCGCTCTGAGTTGCTCCGTAAATTGACCGAAACGTTCGGCTCGTGGTAACAGCCTAAAGGTGGCACTGATATGTTCTGCCATCCGCGAACAGGTAAGCCACTACCTAAATACCCTCGCATTAAGACACCTAAAGTTGGTGGTATCTTTAAGAAGCCTAAGAACAAGGCACAGCGAGAAGGCCGTGAGCCTTGCGAACTTGATACCCGCGAGTACGTTGCTGGTGCTCCTTACAcccc                                                    <  1:793979/301‑1 (MQ=255)`  
                                                                                                                                                                                                                                                `gcgcAACGGGTTCCCATTTGACACAAAAGCAATCGAAGAGTTGTACGTAGAGTTAGCTTCTCGCCACTCTGAGTTGCTCCGTAAATTGACCGAAACGTTCGGCTCGTGGTATCAGCCTAAAGGTGGCACTGAGATGTTCTGCCATCCGCGAACAGGTAAGCCACTACCTAAATACCATCGCATTAAGACACCTAAAGTTGGTGGTATCTTTAAGAAGCCTAAGAACAAGGCACAGCGAGAAGGCCGTGAGCCTTGCGAACTTGATACCCGCGAGTACGTTGCTGGTGCTCCtgtctcttat                                                   >  1:60284/1‑292 (MQ=255)`  
                                                                                                                                                                                                                                                       `ctcggagatgtgtataagagacagaTCGAAGAGTTGTACGTAGAGTTAGCTTCTCGCCGCTCTGCGTTGCTCCGTAAATTGACCGAAACTTTCTGCTCGTGGTATCAGCCTAAAGGTGGCACTGAGATGTTCTGCCATCCGCGAACAGGTAAGCCACTACCTAAATACCCTCGCATTAAGACACCTAAAGTTGGTGGTATCTTTAAGAAGCCTAAGAACAAGGCACAGCGAGAAGGCCGTGAGCCTTGCGAACTTGATACCCGCGAGTACGTTGCTGGTGCTCCTTACACACCAGTTGAAc                                            <  1:724808/277‑1 (MQ=255)`  
                                                                                                                                                                                                                                                                `tttGACACAAAAGCAATCGAAGAGTTGTACGTAGAGTTAGCTTCTCGCCGCTCTGAGTTGCTCCGTAAATTGACCGAAACGTTCGGCTCGTGGTATCAGCCTAAAGGTGGCACTGAGATGTTCTGCCATCCGCGAACAGGTAAGCCACTACCTAAATACCCTCTCATTAAGACACCTAAAGTTGGTGGTATCTTTAAGAAGCCTAAGAACAAGGCACAGCGAGAAGGCCGTGAGCCTTGCGAACTTGATACCCGCGAGTACGTTGCTGGTGCTCCTTACACCCCAGTTGAACATGTTgtgt                                   <  1:777838/301‑1 (MQ=255)`  
                                                                                                                                                                                                                                                                       `aaaaaGCAATCGAAGAGTTGTACGTAGGGTTAGCTTCTCGCCGCGCTGAGTTGCTCCGTAAATTGACCGAAACGTTCGGCTCGGGGTATCAGCCTAAAGGTGGCACTGAGATGTTCTGCCATCCGCGAACAGGTAAGCCACTACCTAAATAGCCTCGCATTAAGACACCTAAAGTTGGTGGTATCTTTAAGAAGCCTAAGAACAAGGCACAGCGAGAAGGCCGTGAGCCTTGCGAACTTGATACCCGCGAGTACGTTGCTGGTGCTCCTTACACCCCAGTTGAACATGTTGTGTTTAGccc                            <  1:37735/300‑1 (MQ=255)`  
                                                                                                                                                                                                                                                                               `aTCGAAGAGTTGTACGTAGAGTTAGCTTCTCGCCGCTCTGAGTTGCTCCGTAAATTGACCGAAACGTTCGGCTCGTGGTATCAGCCTAAAGGTGGCACTGAGATGTTCTGCCATCCGCGAACAGGTAAGCCACTACCTAAATATCCTCGCATTAAGACACCTAAAGTTGGTGGTATCTTTAAGAAGCCTAAGAACAAGGCACAGCGAGAAGTCCGTGAGCCTTGCGAACTTGATACCCGCGAGTACGTTGCTGGTGCTCCTTACACCCCAGTTGAACATGTTGTGTTTAACCCTTCGtctc                    >  1:734672/1‑301 (MQ=255)`  
                                                                                                                                                                                                                                                                               `aTCGAAGAGTTGTACGTAGAGTTAGCTTCTCGCCGCTCTGAGTTGCTCCGTAAATTGACCGAAACGTTCGGCTCGTGGTATCAGCCTAAAGGTGGCACTGAGATGTTCTGCCATCCGCGAACAGGTAAGCCACTACCTAAATACCCTCGCATTAAGACACCTAAAGTTGGTGGTATCTTTAAGAAGCCTAAGAACAAGGCACAGCGAGAAGGCCGTGAGCCTTGCGAACTTGATACCCGCGAGTACGTTGCTGGTGCTCCTTACACCCCAGTTGAACAtgtctgtctcttatacacatttc                    >  1:51045/1‑281 (MQ=255)`  
                                                                                                                                                                                                                                                                               `aTCGAAGAGTTGTACGTAGAGTTAGCTTCTCGCCGCTCTGAGTTGCTCCGTAAATTGACCGAAACGTTCGGCTCGTGGTATCAGCCTAAAGGTGGCACTGAGATGTTCTGCCATCCGCGAACAGGTAAGCCACTACCTAAATACCCTCGCATTAAGACACCTAAAGTTGGTGGTATCTTTAAGAAGCCTAAGAACAAGGCACAGCGAGAAGGCCGTGAGCCTTGCGAACTTGATACCCGCAAGTACGTTGCTGGTGCTCCTTACAACCCAGTTGAACATGTTGTGTTTAACCCTTCGtctc                    >  1:101024/1‑301 (MQ=255)`  
                                                                                                                                                                                                                                                                                     `gagacagaCGTAGAGTTAGCTTCTCGCCGCTCTGAGTTGCTACGTAAATTGACCGAAACGTTCGGCTCGTGGTATCAGCCTAAAGGTGGCACTGAGATGTTCTGCCATCCGCGAACAGGTAAGCCACTACCTAAATACCCTCGCATTAAGACACCTAAAGTTGGTGGTATATTTAAGAAGCCTAAGAACAAGGCACAGCGAGAAGGCCGTGAGCCTTGCGAACTTGATACCCGCGAGTACGTTGCTGGTGCTCCTTACACCCCAGTTGAACATGTTGTGTTTAACCATTCGTCTCGTGAcc              <  1:454301/294‑1 (MQ=255)`  
                                                                                                                                                                                                                                                                                     `gagTTGTACGTAGAGTTAGCTTCTCGCCGCGCTGAGTTGCTCCGTAAATTGACCGAAACGGTCGGCTCGTGGTATCAGCCTAAAGGTGGCACTGAGATGTTCTGCCATCCGCGAACAGGTAAGCCACTACCTAAATACCCTCGCATTAAGACACCTAAAGTTGGTGGTATCTTTAAGAAGCCTAAGAACAAGGCACAGCTAGAAGGCCGTGAGCCTTGCGAACTTGATACCCGCGAGTACGTTGCTGGTGCTCCTTACACCACAGTTGAACATGTTGTGTTTAACCATTCGTCTCGTGAcc              <  1:217031/301‑1 (MQ=255)`  
                                                                                                                                                                                                                                                                                       `gTTGTACGTAGAGTTAGCTTCTCGCCGCTCTGAGTTGCTCCGTAAATTGACCGAAACGTTCGGCTCGTGGTATCAGCCTAAAGGTGGCACTGAGATGTTCTGCCATCCGCGAACAGGTAAGCCACTACCTAAATACCCTCGCATTAAGACACCTACAGTTGGTGGTATCTTTAAGAAGCCTAAGAACAAGGCACAGCGAGAAGGCCGTGAGCCTTGCGAACTTGATACCCGCGAGTACGTTGCTGGTGCTCCTTACACCCCAGTTGTACATGTTGTGTTTAACCATTCGtctctgtctctt            >  1:708103/1‑293 (MQ=255)`  
                                                                                                                                                                                                                                                                                          `gTACGTAGAGTTAGCTTCTCGCCGCTCTGAGTTGCTCCGTAAATTGACCGAAACGTTCGGCTCGTGGTATCAGCCTAAAGGTGGCACTGAGATGTTCTGCCATCCGCGAACAGGTAAGCCACTACCTAAATACCCTCGCATTAAGACACCTAAAGTTGGTGGTATCTTTAAGAAGCCTAAGAACAAGGCACAGCGAGAAGGCCGGGAGCCTTGCGAACTTGATACCCGGGAGTACGTAGCTGGTGCCACTTCCACCCCAGTTGAACAGGTTTTGATTAACCTTTCGTCCCGTGACCAAAtg         >  1:470254/1‑300 (MQ=255)`  
                                                                                                                                                                                                                                                                                              `ttAGTGTTAGCTTCTCGCCGCTCTGAGTTGCTCCGTAAATTGACCGCAACGTTCGGTTCGTGGTATCAGCCTAAAGGTGTCACTGAGATGTTCTGCCATCCGCGAACAGGTAAGCCACTACCTAAATACCCTCGCATTAAGACACCTAAAGTTGGTGGTATCTTTAAGAATCCTAAGAACAAGGCACAGCGAGAAGGCCGTGAGCCTTGCGAACTTGATACCCGCGAGTACGTTGCTGGTGCTCCTTACACCCCAGTTGAACATGTTGTGTTTAACCCTTCGTCTCGTGACCACATTCaga     <  1:185405/300‑1 (MQ=255)`  
                                                                                                                                                                                                                                                                                                `agagTTAGCTTCTCGACGCTCTGAGTTGCTCCGTAAATTGACCGAAACGTTCGGCTCGTGGTATCAGCCTAAAGGTGGCACTGAGATGTTCTGCCATCCGCGAACAGGTAAGCCACTACCTAAATACCCTCGCATTAAGACACCTAAAGTTGGTGGTATCTTTAAGAAGCCTAAGAACAAGGCACAGCGAGAAGGCCGTGAGCCTTGCGAACTTGATACCCGCGAGTACGTTGCTGGTGCTCCTTACACCCCAGTTGAACATGTTGTGTTTAACACTTCGTCTCGTGACCACATTCagaag   <  1:524524/301‑1 (MQ=255)`  

                                                                                                                                                                                                                                                                                                          `|`                                                                                                                                                                                                                                                                                                     
`AGAGCAGGGTGAAGAATACGTTGACGGAATGGAGTGGTGGAACTTCAACGAAGAGATGATGGACTATAACGTTCAGGACGTTGTGGTAACTAAAGCTCTCCTTGAGAAGCTACTCTCTGACAAACATTACTTCCCTCCTGAGATTGACTTTACGGACGTAGGATACACTACGTTCTGGTCAGAATCCCTTGAGGCCGTTGACATTGAACATCGTGCTGCATGGCTGCTCGCTAAACAAGAGCGCAACGGGTTCCCGTTTGACACAAAAGCAATCGAAGAGTTGTACGTAGAGTTAGCTGCTCGCCGCTCTGAGTTGCTCCGTAAATTGACCGAAACGTTCGGCTCGTGGTATCAGCCTAAAGGTGGCACTGAGATGTTCTGCCATCCGCGAACAGGTAAGCCACTACCTAAATACCCTCGCATTAAGACACCTAAAGTTGGTGGTATCTTTAAGAAGCCTAAGAACAAGGCACAGCGAGAAGGCCGTGAGCCTTGCGAACTTGATACCCGCGAGTACGTTGCTGGTGCTCCTTACACCCCAGTTGAACATGTTGTGTTTAACCCTTCGTCTCGTGACCACATTCAGAAGA  >  NC_001604.1/14796‑15385`

  

Alignment Legend

Aligned base mismatch/match (shaded by quality score): `ATCG/ATCG` `< 3 ≤` `ATCG/ATCG` `< 11 ≤` `ATCG/ATCG` `< 25 ≤` `ATCG/ATCG` `< 36 ≤` `ATCG/ATCG` `< 38 ≤` `ATCG/ATCG`

Unaligned base: `atcg`    Masked matching base: `atcg`    Alignment gap: `‑`    Deleted base: `‑`
